# The reliability of replications: a study in computational reproductions

**DOI:** 10.1098/rsos.241038

**Published:** 2025-03-19

**Authors:** Nate Breznau, Eike Mark Rinke, Alexander Wuttke, Muna Adem, Jule Adriaans, Esra Akdeniz, Amalia Alvarez-Benjumea, Henrik K. Andersen, Daniel Auer, Flavio Azevedo, Oke Bahnsen, Ling Bai, Dave Balzer, Paul C. Bauer, Gerrit Bauer, Markus Baumann, Sharon Baute, Verena Benoit, Julian Bernauer, Carl Berning, Anna Berthold, Felix S. Bethke, Thomas Biegert, Katharina Blinzler, Johannes N. Blumenberg, Licia Bobzien, Andrea Bohman, Thijs Bol, Amie Bostic, Zuzanna Brzozowska, Katharina Burgdorf, Kaspar Burger, Kathrin Busch, Juan-Carlos Castillo, Nathan Chan, Pablo Christmann, Roxanne Connelly, Christian S. Czymara, Elena Damian, Eline A. de Rooij, Alejandro Ecker, Achim Edelmann, Christina Eder, Maureen A. Eger, Simon Ellerbrock, Anna Forke, Andrea Forster, Danilo Freire, Chris Gaasendam, Konstantin Gavras, Vernon Gayle, Theresa Gessler, Timo Gnambs, Amélie Godefroidt, Max Grömping, Martin Groß, Stefan Gruber, Tobias Gummer, Andreas Hadjar, Verena Halbherr, Jan Paul Heisig, Sebastian Hellmeier, Stefanie Heyne, Magdalena Hirsch, Mikael Hjerm, Oshrat Hochman, Jan H. Höffler, Andreas Hövermann, Sophia Hunger, Christian Hunkler, Nora Huth-Stöckle, Zsófia S. Ignácz, Sabine Israel, Laura Jacobs, Jannes Jacobsen, Bastian Jaeger, Sebastian Jungkunz, Nils Jungmann, Jennifer Kanjana, Mathias Kauff, Salman Khan, Sayak Khatua, Manuel Kleinert, Julia Klinger, Jan-Philipp Kolb, Marta Kołczyńska, John Kuk, Katharina Kunißen, Dafina Kurti Sinatra, Alexander Langenkamp, Robin C. Lee, Philipp M. Lersch, David Liu, Lea-Maria Löbel, Philipp Lutscher, Matthias Mader, Joan E. Madia, Natalia Malancu, Luis Maldonado, Helge Marahrens, Nicole Martin, Paul Martinez, Jochen Mayerl, Oscar J. Mayorga, Robert McDonnell, Patricia McManus, Kyle McWagner, Cecil Meeusen, Daniel Meierrieks, Jonathan Mellon, Friedolin Merhout, Samuel Merk, Daniel Meyer, Leticia Micheli, Jonathan Mijs, Cristóbal Moya, Marcel Neunhoeffer, Daniel Nüst, Olav Nygård, Fabian Ochsenfeld, Gunnar Otte, Anna Pechenkina, Mark Pickup, Christopher Prosser, Louis Raes, Kevin Ralston, Miguel Ramos, Frank Reichert, Arne Roets, Jonathan Rogers, Guido Ropers, Robin Samuel, Gregor Sand, Constanza Sanhueza Petrarca, Ariela Schachter, Merlin Schaeffer, David Schieferdecker, Elmar Schlueter, Katja Schmidt, Regine Schmidt, Alexander Schmidt-Catran, Claudia Schmiedeberg, Jürgen Schneider, Martijn Schoonvelde, Julia Schulte-Cloos, Sandy Schumann, Reinhard Schunck, Julian Seuring, Henning Silber, Willem Sleegers, Nico Sonntag, Alexander Staudt, Nadia Steiber, Nils D. Steiner, Sebastian Sternberg, Dieter Stiers, Dragana Stojmenovska, Nora Storz, Erich Striessnig, Anne-Kathrin Stroppe, Jordan W. Suchow, Janna Teltemann, Andrey Tibajev, Brian Tung, Giacomo Vagni, Jasper Van Assche, Meta van der Linden, Jolanda van der Noll, Arno Van Hootegem, Stefan Vogtenhuber, Bogdan Voicu, Fieke Wagemans, Nadja Wehl, Hannah Werner, Brenton M. Wiernik, Fabian Winter, Christof Wolf, Cary Wu, Yuki Yamada, Björn Zakula, Nan Zhang, Conrad Ziller, Stefan Zins, Tomasz Żółtak, Hung H.V. Nguyen

**Affiliations:** ^1^ Organization and Program Planning, German Institute for Adult Education—Leibniz Center of Lifelong Learning, Bonn 53175, Germany; ^2^ School of Politics and International Studies, University of Leeds, Leeds LS2 9JT, UK; ^3^ Geschwister Scholl Institute, LMU Munich, Munich 81541, Germany; ^4^ Department of Sociology, University of Maryland, College Park 47405, USA; ^5^ Faculty of Sociology, University of Bielefeld, Bielefeld 33615, Germany; ^6^ School of Medicine, Marmara University, Istanbul 34722, Turkey; ^7^ The Institute of Public Goods and Policies (IPP), Centro de Ciencias Humanas y Sociales—Consejo Superior de Investigaciones Científicas, Madrid 28003, Spain; ^8^ Institute of Sociology, Chemnitz University of Technology, Chemnitz 09126, Germany; ^9^ Social and Political Science, Collegio Carlo Alberto, Turin 10122, Italy; ^10^ Department of Interdisciplinary Social Science, Utrecht University, Utrecht 3584CH, Netherlands; ^11^ School of Social Sciences, University of Mannheim, Mannheim 68159, Germany; ^12^ Institute of Sociology, Johannes Gutenberg University Mainz, Mainz 55128, Germany; ^13^ Institute of Political Science, University of Freiburg, Freiburg im Breisgau 79085, Germany; ^14^ Institute of Statistics, LMU Munich, Munich, 79098, Germany; ^15^ Department of Sociology, LMU Munich, Munich 80801, Germany; ^16^ Office of the Executive Board, GESIS—Leibniz Institute for the Social Sciences, Mannheim 68159, Germany; ^17^ Department of Politics and Public Administration, University of Konstanz, Konstanz 78457, Germany; ^18^ Faculty of Social Sciences, Economics, and Business Administration, University of Bamberg, Bamberg 96052, Germany; ^19^ Mannheim Centre for European Social Research (MZES), University of Mannheim, Mannheim 68131, Germany; ^20^ Institute for Political Science, Johannes Gutenberg University Mainz, Mainz 55099, Germany; ^21^ Research Depart­ment IV: Intrastate Conflict, Peace Research Institute Frankfurt (PRIF), Frankfurt 60329, Germany; ^22^ Department of Social Policy, London School of Economics and Political Science, London WC2A 2AE, UK; ^23^ Survey Data Curation, GESIS—Leibniz Institute for the Social Sciences, Mannheim 50667, Germany; ^24^ Knowledge Exchange and Outreach, GESIS—Leibniz-Institute for the Social Sciences, Mannheim 67067, Germany; ^25^ Faculty of Economics and Social Sciences, University of Potsdam, Potsdam 10117, Germany; ^26^ Department of Sociology, Umeå University, Umeå 901 87, Sweden; ^27^ Department of Sociology, University of Amsterdam, Amsterdam 1012 WP, Netherlands; ^28^ Department of Sociology, The University of Texas Rio Grande Valley, Edinburg TX 78539, USA; ^29^ Vienna Institute of Demography, Austrian Academy of Sciences, Vienna, Austria & Austrian National Public Health Institute (Gesundheit Österreich GmbH, GÖG), Vienna, Austria; ^30^ School of Social Sciences, University of Bremen, Bremen 28359, Germany; ^31^ Department of Education, University of Potsdam, Potsdam, 14469, Germany; ^32^ Jacobs Center for Productive Youth Development, University of Zurich, Zürich, 8006, Switzerland; ^33^ Social Research Institute, University College London, London WC1H 0AL, UK; ^34^ Independent Researcher; ^35^ Department of Sociology Universidad de Chile, Millennium Nucleus on Digital Inequalities and Opportunities (NUDOS NCS2022_046), and Center for Social Conflict and Cohesion Studies (COES), University of Chile, Santiago 7800284, Chile; ^36^ Department of Political Science and International Relations, Loyola Marymount University, Los Angeles CA 90045, USA; ^37^ Data and Research on Society, GESIS—Leibniz-Institute for the Social Sciences, Mannheim 68159, Germany; ^38^ School of Social and Political Science, University of Edinburgh, Edinburgh EH8 9LD, Scotland; ^39^ Institute of Sociology, Goethe-University Frankfurt, Frankfurt 60323, Germany; ^40^ Epidemiology and Public Health, Sciensano, Brussels 1050, Belgium; ^41^ Political Science, Simon Fraser University, Burnaby V5A 1S6, Canada; ^42^ Institute of Political Science, Heidelberg University, Heidelberg 69115, Germany; ^43^ Médialab Sciences Po, Paris 75007, France; ^44^ Department of Sociology, Umeå University, Umeå 901 87, Sweden; ^45^ Center for Advanced Study in the Behavioral Sciences, Stanford, CA 94305, USA; ^46^ Department of Sociology, Utrecht University, Utrecht 3584 CS, Netherlands; ^47^ Department of Quantitative Theory and Methods, Emory University, Atlanta 30306, USA; ^48^ Department of Work and Social Economy, Government of Flanders-Belgium,; ^49^ Kulturwissenschaftliche Fakultät, Europa Universität Viadrina, Frankfurt 15230, Germany; ^50^ Educational Measurement, Leibniz Institute for Educational Trajectories, Bamberg 96047, Germany; ^51^ Centre for Research on Peace and Development, KU Leuven, Leuven 3000, Belgium; ^52^ School of Government and International Relations, Griffith University, Nathan, Queensland 4111, Australia; ^53^ Department of Sociology, University of Tübingen, Tübingen 72074, Germany; ^54^ Research Data Center and Communication, SHARE BERLIN Institute, Berlin 10115, Germany; ^55^ Data and Research on Society, GESIS—Leibniz Institute for the Social Sciences, Mannheim 68159, Germany; ^56^ Division Sociology, Social Policy and Social Work, University of Fribourg, Fribourg CH-1700, Switzerland; ^57^ Association for Doctoral Studies Baden-Wuerttemberg 70174, Germany; ^58^ Research Group ‘Health and Social Inequality’, WZB Berlin Social Science Center, Berlin 10785, Germany; ^59^ Transformations of Democracy Unit, WZB Berlin Social Science Center, Berlin 10785, Germany; ^60^ Research Unit Migration, Integration, Transnationalization, WZB Berlin Social Science Center, Berlin 10785, Germany; ^61^ Data and Research on Society, GESIS—Leibniz Institue for the Social Sciences, Mannheim 68159, Germany; ^62^ Facultad de Emprendimiento, Negocios y Economía, Universidad Espíritu Santo, ReplicationWiki, and EQ-Lab,; ^63^ Wirtschafts- und Sozialwissenschaftliches Institut (WSI), Hans-Böckler-Foundation, Düsseldorf 40474, Germany; ^64^ SOCIUM—Research Center on Inequality and Social Policy, University of Bremen, Bremen 10785, Germany; ^65^ Berlin Institute for Integration and Migration Research (BIM), Humboldt Universität zu Berlin, Berlin 10099, Germany; ^66^ School of Human and Social Sciences, University of Wuppertal, Wuppertal 42119, Germany; ^67^ Department of Political Science, University of Antwerp, Antwerpen 2000, Belgium; ^68^ Cluster ‘Data-Methods-Monitoring’, German Center for Integration and Migration Research (DeZIM),; ^69^ Department of Social Psychology, Tilburg University, Tilburg 5037AB, Netherlands; ^70^ Institute of Political Science and Sociology, University of Bonn, Bonn 53111, Germany; ^71^ Survey Data Curation, GESIS—Leibniz Institute for the Social Sciences, Mannheim 50667, Germany; ^72^ Department of Psychology, Medical School Hamburg, Hamburg 20457, Germany; ^73^ Economics, University of Illinois, Chicago, Chicago, IL, USA; ^74^ School of Public Policy, Oregon State University, Corvallis 97330, USA; ^75^ Institute of Sociology, Justus Liebig University Giessen, Giessen 35394, Germany; ^76^ Statistisches Bundesamt, Statistisches Bundesamt Wiesbaden, Wiesbaden 67549, Germany; ^77^ Department of Research on Social and Institutional Transformations, Institute of Political Studies of the Polish Academy of Sciences, Warsaw 00-625, Poland; ^78^ Department of Political Science, Michigan State University, East Lansing 48823, USA; ^79^ Center for Evaluation, Independent Researcher (Formerly Uni Cologne),; ^80^ Department of Sociology, Princeton University, Princeton, USA; ^81^ Socio-Economic Panel, German Institute for Economic Research, Berlin 10117, Germany; ^82^ Socio-Economic Panel, German Institute for Economic Research, Berlin 10117, Germany; ^83^ Department of Political Science, University of Oslo, Oslo 0851, Norway; ^84^ Department of Philosophy, Politics and Economics, Witen/Herdecke University, Witten 58488, Germany; ^85^ Department of Primary Care and Health Sciences, University of Oxford, Oxford OX11JD, England; ^86^ Swiss Forum for Migration and Population Studies, University of Neuchatel, Neuchâtel 1205, Switzerland; ^87^ Instituto de Sociologia, Pontifical Catholic University of Chile, Santiago 7820436, Chile; ^88^ Massive Data Institute, Georgetown University, Washington D.C. 20057, USA; ^89^ Department of Politics, University of Manchester, Manchester M19JS, UK; ^90^ Department of Institutional Research, Western Governors University, Millcreek 84107, USA; ^91^ Director of Data for Freedom, Equity Research Cooperative, 19107; ^92^ Department of Sociology, Indiana University Bloomington, Bloomington, IN 47405, USA; ^93^ Department of Political Science, University of Wisconsin-Milwaukee, Milwaukee, WI 53211, USA; ^94^ Department of Sociology, Center for Sociological Research, KU Leuven, 3000; ^95^ Department of Politics, Westpoint Department of Systems Engineering, M19 2JS; ^96^ Department of Sociology and Centre for Social Data Science, University of Copenhagen, 1353; ^97^ Department of School Development, Karlsruhe University of Education, Karlsruhe 76133, Germany; ^98^ Competence Centre for Regional Development, Federal Institute for Research on Building, Urban Affairs and Spatial Development (BBSR), 03048; ^99^ Department of Social, Economic & Organisational Psychology, Leiden University, Leiden 2333AK, The Netherlands; ^100^ Department of Sociology, Boston University, Boston, MA 02215, USA; ^101^ Socio-Economic Panel, Institute for Economic Research, Berlin 10117, Germany; ^102^ School of Social Sciences, LMU Munich, Munich, Germany; ^103^ Department of Geosciences, Technische Universität Dresden, Dresden 01069, Germany; ^104^ Division of Migration, Ethnicity and Society (REMESO), Linköping University, 60174; ^105^ Administrative Headquarters, Max Planck Society, Munich 80539, Germany; ^106^ Department of Political Science, Utah State University, Logan, UT 84321, USA; ^107^ Political Science, Simon Fraser University, Canada; ^108^ Department of Economics, Tilburg University, Tilburg 5037 AB, The Netherlands; ^109^ Department of Social Policy, Sociology and Criminology, University of Birmingham, Birmingham B15 2TT, UK; ^110^ Department of Developmental, Personality, and Social Psychology, Ghent University, Sint-Pietersnieuwstraat, B-9000, Belgium; ^111^ School of Law, Empirical Research Group, University of California, Los Angeles, Los Angeles, CA, USA; ^112^ Department of Social Sciences, University of Luxembourg, 4366, Luxembourg; ^113^ SHARE Operations, SHARE Berlin Institute, Berlin 10115, Germany; ^114^ School of Politics and International Relations, Australian National University, Canberra 2132, Australia; ^115^ Department of Sociology, Washington University in St. Louis, St. Louis MO 63130, USA; ^116^ Institute for Media and Communication Studies, Freie Universität Berlin, Berlin 14195, Germany; ^117^ Department of Social Sciences, Humboldt University Berlin, Berlin, 10117, Germany; ^118^ Department of Social Sciences, Socio-Economic Panel, Berlin, 10117, Germany; ^119^ Teacher and Teaching Quality, Leibniz Institute for Research and Information in Education, Frankfurt 60323, Germany; ^120^ Chair group European Politics and Society, University of Groningen, Groningen 9712 EK, Netherlands; ^121^ Department of Political Science, University of Marburg, Marburg 35037, Germany; ^122^ Department of Security and Crime Science, University College London, London WC1E 6BT, UK; ^123^ School of Human and Social Sciences, University of Wuppertal, Wuppertal 42119, Germany; ^124^ Department Migration, Leibniz Institute for Educational Trajectories, Bamberg 96047, Germany; ^125^ Institute for Social Research, University of Michigan, Ann Arbor MI 48109, USA; ^126^ Department of Sociology, University of Vienna, Vienna 1090, Austria; ^127^ Centre for Political Science Research, KU Leuven, Leuven 3000, Belgium; ^128^ Expert Council on Intergration and Migration, Berlin 10178, Germany; ^129^ School of Business, Stevens Institute of Technology, Hoboken 07030, USA; ^130^ Institute for Social Sciences, University of Hildesheim, Hildesheim 31141, Germany; ^131^ Department of Women's and Children's Health, Uppsala University, Uppsala SE-751 05, Sweden; ^132^ Department of Sociology, Washington University in St. Louis, St. Louis 63130, USA; ^133^ Social Research Institute (UCL), University College London, London WC1E 6BT, USA; ^134^ Center for Social and Cultural Psychology (CESCUP), Université Libre de Bruxelles, Brussels, BE-1050, Belgium; ^135^ Optentia Research Unit, North-West University, Potchefstroom, 2531, South Africa; ^136^ Department of Psychology, University of Hagen, Hagen 58097, Germany; ^137^ Department of Sociology and Human Geography, University of Oslo, Oslo 851, Norway; ^138^ Education and Employment, Institute for Advanced Studies, Vienna 1080, Austria; ^139^ Research Institute for Quality of Life, Romanian Academy, Bucharest, 010071, Romania; ^140^ Department of Sociology, Lucian Blaga University of Sibiu, Sibiu, 550024, Romania; ^141^ Beleidsvisies, Burgervisies en Gedragingen (Policy Perspectives, Citizen Perspectives, and Behaviors), Netherlands Institute for Social Research, The Hague 2594, Netherlands; ^142^ Research Cluster ‘The Politics of Inequality’, University of Konstanz, Konstanz 78464, Germany; ^143^ Department of Political Science, University of Zurich, Zürich 8050, Switzerland; ^144^ Mechanisms of Normative Change, Max-Planck-Institute for Research on Collective Goods, Bonn 53113, Germany; ^145^ GESIS Leibniz-Institute for the Social Science & University of Mannheim, Mannheim 68159, Germany; ^146^ Department of Sociology, York University, Toronto M3J 1P3, Canada; ^147^ Faculty of Arts and Science, Kyushu University, Fukuoka 819-0395, Japan; ^148^ Department of Political Science, University of Duisburg-Essen, Duisburg-Essen 47057, Germany; ^149^ Institute for Employment Research, Federal Employment Agency, Nuremberg 90478, Germany; ^150^ Department of Computational Social Sciences, Institute of Philosophy and Sociology of the Polish Academy of Sciences, Warsaw 00-330, Poland; ^151^ Political Science, University of Bremen, Bremen 28359, Germany

**Keywords:** reliability, replications, computational reproduction, social and behavioural sciences

## Abstract

This study investigates researcher variability in computational reproduction, an activity for which it is least expected. Eighty-five independent teams attempted numerical replication of results from an original study of policy preferences and immigration. Reproduction teams were randomly grouped into a ‘transparent group’ receiving original study and code or ‘opaque group’ receiving only a method and results description and no code. The transparent group mostly verified original results (95.7% same sign and *p*-value cutoff), while the opaque group had less success (89.3%). Second-decimal place exact numerical reproductions were less common (76.9 and 48.1%). Qualitative investigation of the workflows revealed many causes of error, including mistakes and procedural variations. When curating mistakes, we still find that only the transparent group was reliably successful. Our findings imply a need for transparency, but also more. Institutional checks and less subjective difficulty for researchers ‘doing reproduction’ would help, implying a need for better training. We also urge increased awareness of complexity in the research process and in ‘push button’ replications.

## 1. Introduction

A basic requirement of science being reliable is computational reproducibility [1]: the capacity ‘for assessing the value or accuracy of scientific claims based on the original methods, data and code’ [2]. Computational reproduction is a special case of scientific reliability checking because it involves no research design decision-making. There is no need to specify methods, empirical research questions or define estimands [3,4]. Moreover, the data are pre-existing and ostensibly identical. Computationally reproducing existing numerical results should thus be straightforward, yet recent findings in meta-science suggest this is often not the case. Computational reproductions are subject to uncertainty resulting from the intransparency of an original study; sometimes what should be identical data varies because of read-in software or version changes. Also, idiosyncrasies across researchers might lead them to process the data in ways that cause different values in the computing environment. In this study, we look at computational reproducibility via an experiment in which 85 teams of 1–3 researchers were randomly split into two groups with more or less access to replication materials from a published study. They were asked to replicate the numerical results of the original study using the same starting data and same methods. We observed these researchers with the goal of understanding the reliability of computational reproductions, and identifying the sources of alarmingly high uncertainty found in other reproducibility studies.

Reproducibility is currently an intense topic in the academic community [5–7]. The practice of public code sharing is essential for reproducibility, but access to others’ entire research pipelines is still somewhat of a pipe dream in many social and behavioural sciences. In a recent survey of active researchers, only 18% in social science (*n* = 733) and 17% in business and economics (*n* = 592) provided code for their published statistical results [8].^1^ Another study found that of all social science publications in the journals *Science* and *Nature* between 2000 and 2019, only 20% came with reproducible materials (usually data and code), and this only increased to 40% when the authors were contacted personally [9]. In a similar vein, only 38% of authors from over a thousand studies using data from the *European Social Survey* shared their code after receiving a request [10]. Although a recent study shows that social and behavioural scientists overwhelmingly support code sharing, evidence suggests that in more than half of studies it is not practised [11].

Code sharing alone does not solve all problems of reproducibility. Even with access to replication materials, computational replication regularly fails [12–14]. For example, the *American Journal of Political Science* (AJPS) started checking the reproducibility of all quantitative research results in papers accepted for publication in 2014. The first 15 studies’ results could not be computationally reproduced with the materials provided, and it often took multiple communications with authors before reproduction was possible [15,16]. In the same vein, many studies attempting to computationally replicate previously published results found striking rates of failure. Hardwicke *et al*. [17] attempted to reproduce the numeric results of 35 studies published in the journal *Cognition,* and even with author assistance, 37% had at least one effect not statistically reproducible within 10% of the original. Stockemer *et al*. [18] failed to reproduce one-third of results among major political behaviour publications in 2015, with one-quarter not producing any numerical results because the code was so poorly organized. More recently, Pérignon *et al*. [19] looked at 168 studies in finance and could reproduce only 52% of the reported numerical effects. These findings demonstrate that there is still much to learn and do before computational reproducibility is the norm.

These previous computational replication attempts demonstrate that verifying the numerical findings of a study is not purely a mechanistic process. It is often possible to achieve replication, but not in a ‘push-button’ format—not without additional communication, materials and support. Although push-button replication is technically possible using virtual computing environments, the skills to build such applications are rare, in particular in the social and behavioural sciences [20]. If push-button replication of numerical research results is only trivially possible on average, this calls into question the current reliability of social and behavioural science at a basic level. The goal of this project is to understand why reproduction fails; hopefully, it holds keys to support developments among academics, journals and institutions seeking to improve the reliability of science.

## 2. Methods

The three principal investigators (PIs) launched this experiment in 2018 with the target of a computational replication of a high-visibility finding from a study using a large multi-level dataset combining survey data and county-level indicators, one requiring relatively strong computational skills [21]. We crowdsourced volunteer teams of a maximum three replicators and observed them as they attempted to verify numerical results from David Brady & Ryan Finnigan’s 2014 article, ‘Does Immigration Undermine Public Support for Social Policy?’ [22]. This article met several criteria: it is highly cited, offers freely available data and code, was independently replicable by two of the study’s PIs in *Stata* and *R* and the original authors consented to the use of their work.

We pre-registered our experimental design and plans to qualitatively code the researchers’ workflows on the Open Science Framework [23]. Power analysis to achieve power of 0.95 under a condition of a small (0.382), medium (0.463) or large (0.518) standardized (Cohen’s d: XY-standardized) difference in average effect size of one group compared to the point estimates of the original study’s findings using a one-tailed 95% confidence interval determined that we need at least 76, 52 or 42 total researchers, respectively. We assumed that we would need only the numerical distance of original and replication results as the outcome variable, but in what follows we present two additional dichotomous measures of a successful replication developed post hoc. We also were unable to imagine in advance all types of errors researchers might make; therefore, our qualitative coding for these emerged directly from the replication teams’ workflows and includes events beyond our theoretical list of predicted mistakes in the pre-registration plan.

All participants were offered co-authorship on the final study if they completed all tasks. Of the initial 105 teams that registered, 99 successfully completed the initial survey. Random assignment of these 99 teams placed 50 into a *transparent group* (TG) that received the Brady & Finnigan article, the original *Stata* code and published technical appendix. The other 49 teams, the *opaque group* (OG), got an anonymized and less transparent version of the study (see electronic supplementary material, appendix A). Comparison of means for team features reveals balanced group assignment (see electronic supplementary material, table S1 in appendix B). Thirteen teams dropped out before starting the replication and one during the replication, leaving 39 teams in the TG and 46 in the OG. All study materials that can be shared publicly are available in our Project Repository.^2^

The Brady & Finnigan study used two waves of *International Social Survey Program* (ISSP) data containing responses to questions about the government’s responsibility to provide various forms of social security and welfare. These data were aggregated to the country-wave level and regressed on stock and flow of immigration measures across different model specifications. To create an intransparent condition of the study for the OG to replicate, the PIs removed two out of six of the dependent variables and the individual-level independent variable measuring income (selected because it had no impact on any effects of interest). The results were presented to the OG in a Methods section written by the PIs describing the models, and direction and significance of coefficients without the original paper, numerical results or code (see electronic supplementary material, appendix A3). Our two experimental conditions were intended to simulate polar extremes in transparency.^3^ For the purpose of simulating a real research endeavour, the participants were instructed to use the software they normally use rather than learn *Stata*. In the TG, the *Stata* users were asked to write their own code based on the *Stata* file from the original authors.^4^

Participants had three weeks to complete the replication, with extensions granted upon request. They were asked to present odds ratios as these were the numerical outcomes reported in the original study. All teams received an Excel template to help standardize reporting. We recorded teams’ numerical reproductions of four dependent variables regressed on different covariates in a total of 26 models (the first four columns of Brady & Finnigan [22] ^5^). Four models included both stock and flow measures of immigration simultaneously (percent foreign-born and net migration), but these were not given to the OG in another step to disguise the original study. Thus, a total of 48 odds ratios in the TG and 40 in the OG were reported for analysis. A few models ran into convergence issues, and a few teams made mistakes that prevented them from arriving at estimates; therefore, not all reported all effects. The final *N* was 3695 odds ratios from 85 teams.

Not all study participants consented to have their names revealed in connection with their code, so we were ethically obligated to redact all identifying features before making it all public (see electronic supplementary material, appendix C). In our research design, we intentionally did not engage in quality control or provide workflow guidance other than the template for reporting results. Some teams submitted in Word or RTF document formats, and others used German-language Excel with commas instead of decimal points. Hence, we constructed a matrix of all results with some inevitable copy-pasting from incompatible file formats. We checked on three independent occasions that their submitted code produced these results. In four teams, parts of the code were missing due to a point-and-click method or researchers not saving their workflows.^6^ To incur minimal ecological bias, we did not demand that these teams produce new code for us. Starting with the teams’ submitted workflows and results, all work conducted for this article, including analysis of teams’ submissions, production of figures and analysis of a participant survey, is available in our Project Repository. In addition to quantifying the uncertainty of computational replications, we qualitatively investigated the content of each team’s workflow to determine the sources of this uncertainty.

Reproducibility, sometimes labelled as computational replication, or computational or analytic reproduction, means obtaining the same results as the original study using the same data and code [2,24]. Practically speaking, this is not always feasible for two reasons. The first is that not all replicators will know how to use or even have access to the software used in an original study, and the second is that different computing environments may produce different levels of decimal place precision by default. Nonetheless, at a basic level, reproducibility should occur at least within a few decimal places and should not depend on the software, so long as identical methods are implemented.

**Figure 1.**
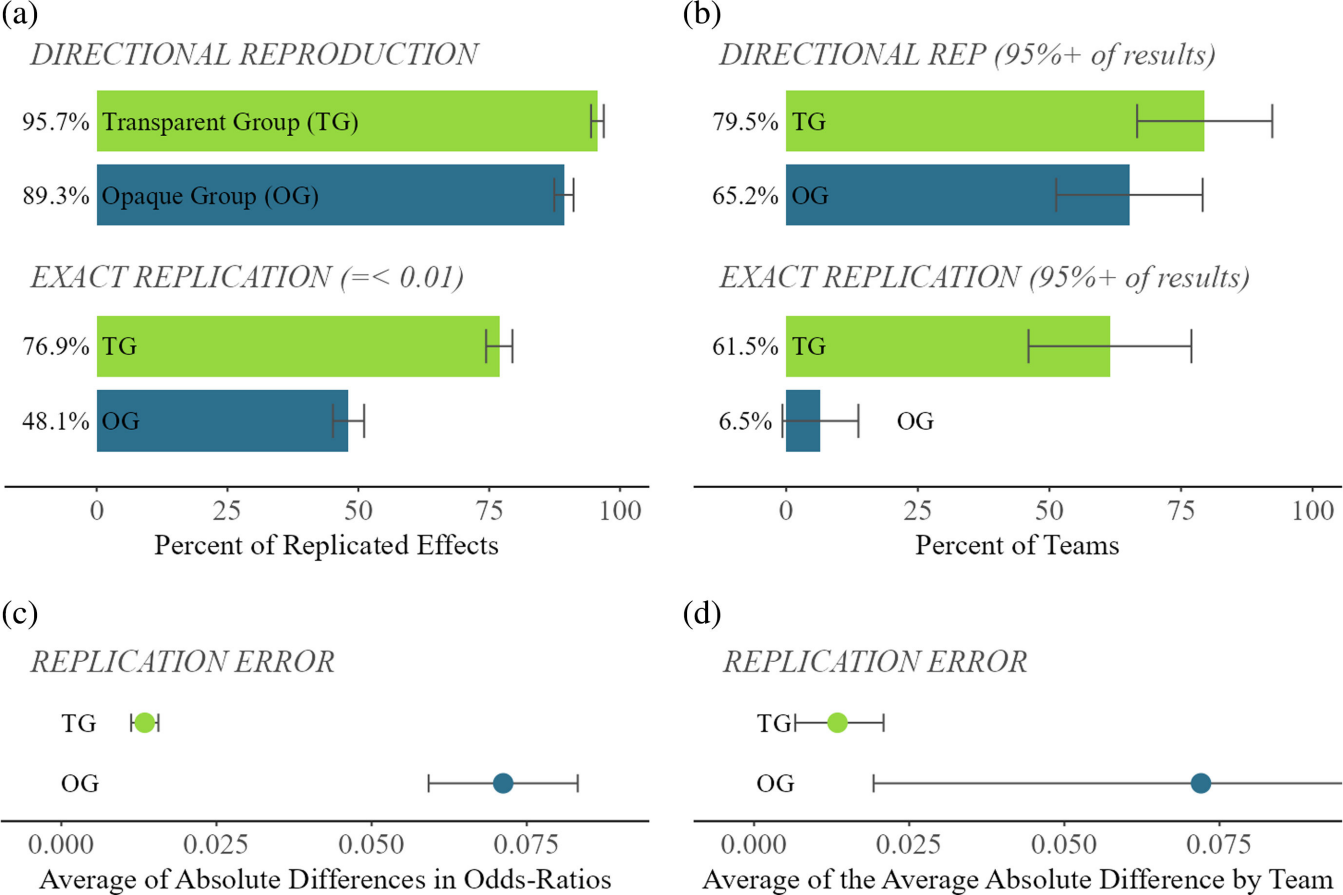
Results of a crowdsourced computational replication from 85 Replicator Teams. Note*:* The study involved a computational reproduction of Brady & Finnigan [22]. Only the Transparent Group “TG” (N = 39 teams & 1,872 effects) had access to the original paper and code, while the Opaque Group “OG” (N = 46 teams & 1,874 effects) had only a description of the methods and no code (see §2 and appendix A). Left-side are effect-level results (a & c) and right-side are team-level results (b & d). Bars represent two-tailed 95% CIs. Differences between experimental groups on all outcome measures are statistically significant at the effect-level with 99% confidence (*p* < 0.01); at the team-level, this is only true of Exact Replication. The between $\sigma^{2}$ (the percentage of the total effect-level variance that occurs between teams, also known as intra-class correlation coefficient [ICC]) are A. DIRECTIONAL REPRODUCTION: TG = 23.9% & OG = 29.4%; B. EXACT REPLICATION: TG = 63.1% & OG = 33.2%; C. REPLICATION ERROR: TG = 39.6% & OG = 93.3%.

Given the uncertainty in the definition of a successful computational replication [25], we developed three different measures to quantify our results. The first we call a *Directional Reproduction*, a dichotomy where results simply point in the same direction and match a null hypothesis significance test that the coefficient is exactly zero with a cut-off of *p* < 0.05 or not. In this scenario, the exact numbers need not match for success. This is important, because the discussion of scientific findings often revolves around the existence of an effect or not. Next, we define a stricter dichotomy of *Exact Replication* where results must be within 1% of the original. This reflects precision, an important aspect of science such that without it, we might not claim reliable results. In this case, our estimand is a numerical odds ratio. Because odds ratios are numerically asymmetric on either side of 1,^7^ we divide original odd ratios by the replicated odds ratio in cases where the replicated odds ratio is smaller than 1 and then multiply by negative one and add one, and we divide the replicated by the original and subtract one in cases where it is larger. Therefore, values within 0.01 (= 1%) are considered an exact replication and comparable in size regardless of signage. The third is a continuous variable capturing *Replication Error* measured as the absolute value of the ratio difference between the replicated odds ratio and original. This was selected for theoretical reasons, as we conceived of the uncertainty of computational reproductions as another plausible estimand that occurs theoretically on a continuum. Rather than success or failure, this points at the reality of science that can be understood as a process of identifying more or less uncertainty. Descriptive statistics of these three measures in raw form are presented in electronic supplementary material, table S1 in appendix B and visualized in figure 1. Further curated and trimmed versions of the results, which we describe shortly, also appear in electronic supplementary material, appendix B.

Using the results from our participant survey, we constructed variables indicating the disciplinary background of the replicator team. In 82 of 85 teams, there was a majority discipline. For the three teams without a majority discipline, we took the discipline of the first team member—the initial responder to the intake survey, and the person responsible for organizing the team. We collapsed this into a variable labelled *Sociology* where sociology = 1 (the largest group of 43 teams) and other disciplines = 0 (political science (22 teams) and a mix of others like psychology, communications, methods-focused degrees and economics). We measured a continuous variable *Stats-Skill* as a latent factor from four questions on team members’ reported experience with statistics.^8^ This variable was particularly important, because it allows us to control for idiosyncratic distribution of skills that were not randomly assigned. We created a variable called *Difficult* scored from 0 to 5 from team-mean responses to the question ‘How difficult did you find the replication task in this first phase?’^9^ Finally, we coded statistical software as *Stata* = 1 (56 teams, the majority) versus other software (= 0, where 22 used *R*, 4 used *SPSS* and 3 used *Mplus*).

**Figure 2.**
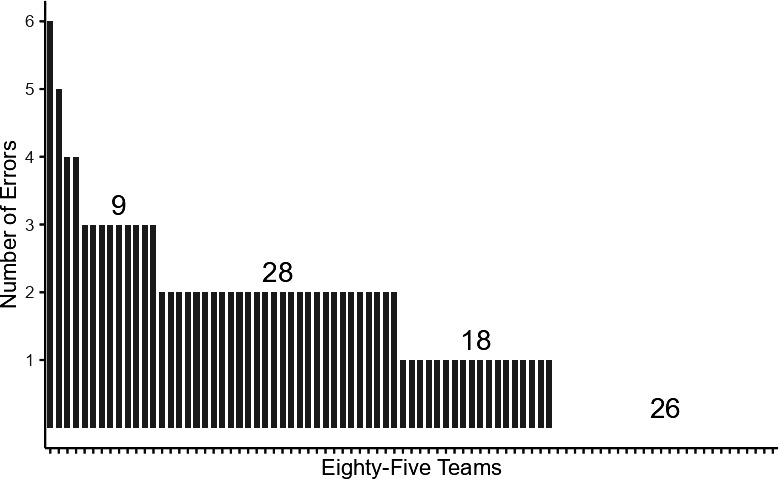
Rates of error per team.

We investigated the sources of researcher variability by qualitatively analysing the content of each team’s code and any comments they provided. Prior to the study, the PIs pre-registered a theoretical set of categories that might be sources of error (see table 1 in [23]); however, grounded in the qualitative content and based on the PI’s knowledge of quantitative research and statistical programming, a total of six coding categories emerged, not all of which were anticipated at the time of pre-registration: Mistake, Procedural, Mistake-Procedural, Missing Component, Interpretational and Questionable Method Knowledge. We present the definitions for each category with selected examples below. A summary of all teams and their category codes is available in electronic supplementary material, table S6 (in appendix B), all original code redacted for identifying features in electronic supplementary material, appendix C^10^ and figure 2 presents the distributions of errors by team.

Because of concerns that the teams’ raw outcomes might lack ecological validity as ‘real-world’ research, we developed a ‘curated’ and a ‘trimmed’ version of the results.

*Curated*: Although not certain, it may be that more errors occurred in our study than in the standard research done by some participants. We expect this because the scientific enterprise is competitive and involves phases of peer review and editorial oversight. Although these vetting procedures do not guarantee that studies are reproducible or reliable, they should cause lower quality work to be published less often and/or motivate researchers to submit higher quality work to pass this vetting process. For curating, we attempted to fix mistakes in a team’s code only if the following two conditions were met: it had to be obvious that it was a mistake and, if so, that we could determine a counterfactual scenario of what the authors would have done instead had they become aware of the mistake. We *only* changed code when we did not have to make *any* decisions. For example, if a team omitted a ‘fixed-effect’ for country or wave, we corrected this. If a team forgot to include a country or added an extra country into the original sample, we adjusted it. However, if we had to make recoding decisions that necessitated choosing from several alternatives, like how to standardize variables across countries or combine categories of employment or education to generate and employment status variable, we did nothing (see detailed coding example in table 2). We left the code untouched if the directional reproduction rate for that team was higher than 95% under the assumption that most social scientists would consider a rate between 95 and 100% a successful test. We corrected code in 14 instances in 12 teams’ workflows. This left us with a set of curated results that *might* better reflect the quality level of the participating researchers under non-experimental conditions. The process of curation was simultaneously a qualitative analysis of each team’s workflow to better understand the causes of computational replication unreliability. We identified far more than our 14 corrected mistakes, but in these cases, we were unable to determine what the team would have done had they been made aware of the mistake.

*Trimmed*: As we show in our qualitative investigation, three teams might not have had the requisite skills or experience to successfully complete a computational reproduction of the original study and have a chance of publication or successful dissemination of results. As this was roughly 5% of the sample, we considered an alternative form of ecological validity where we trimmed 5% of the results that were numerically furthest from the original results. This meant completely dropping two teams’ results from each group.

Using the raw, curated and trimmed results, we employed multivariate regression to identify significant predictors of computational reproducibility (measured as aggregate team average scores on Directional Reproduction, Exact Replication and Replication Error) and the presence of at least one identified Procedural or Mistake error source on the team level as gleaned from our qualitative analysis. Multivariate analysis is only possible at the team level as all independent variables are measured at this level. For the three outcomes, we ran regressions on the raw (table 4 ), trimmed and curated (electronic supplementary material, tables S8 and S9 in appendix B) results averaged by team.

## 3. Results

Figure 1 left column, visualizes the percentage of all effects that were a Directional Reproduction (figure 1a) or Exact Replication (figure 1b), and in the lower panel, it visualizes Replication Error (figure 1*c*) on average by group. Further descriptive statistics are available in electronic supplementary material, tables S1 and S2 in appendix B. The raw effect-level results indicate a high degree of reproducibility with successful directional reproduction in 95.7 and 89.3% of the TG and OG replications, respectively (figure 1a). After curation, these rates jumped to 98.2 and 92.3% (electronic supplementary material, table S1). Pooling results from both experimental conditions yield 92.5% raw, 95.2% curated and 94.1% trimmed rates. The numbers drop for Exact Replications, with pooled results at 62.5, 70.2 and 65.1%, respectively (electronic supplementary material, table S1). In the best-case scenario, with fully transparent materials in the TG, 76.9% of their models were Exact numerical reproductions. Even after correcting obvious mistakes in the TG, the curated results were still only 84.6% of exact reproductions (electronic supplementary material, table S1).

Considering the idea that an entire team or study might be judged as successful or not, the right column figure 1d,e presents results dichotomized so that an entire team is a successful replication only if 95% or more of their models are successful . This suggests that only 79.5% of teams in the TG successfully verified ‘all’ esults from the original study. This was less for teams in the OG, where 62.5% achieved complete numerical directional reproduction of the original study. The curated and trimmed results are only slightly higher (electronic supplementary material, table S1). The difference between experimental groups at the team-level increases dramatically for Exact Replication where 61.5% of the TG had at least 95% of their effects within 0.01 of the original but only 6.5% of teams in the OG; in other words, only 3 out of 46 teams in the OG exactly reproduced the entire set of results from the original study.

Only 14 teams had 100% exact computational reproductions within 1% of the original, and all of them were in the TG. This indicates that most teams committed at least one error (86% overall) in their work—where ‘error’ refers to a failure to achieve an exact computational reproduction. This means that numerical errors at a very high level of precision occur in many teams and may occur in a single model in a team that otherwise successfully replicated all other models. In sum, directional reproductions are relatively consistent independently of the transparency of materials, whereas precision in computational reproduction strongly depends on transparency.

Turning to Replication Error, we find that in the TG both at the effect-level and team-average levels results were within 1% of the original odds ratios on average. Error was dramatically higher in the OG at 32% at the effect level and 33% at the team average level. The density plots below the averages and standard errors show that outliers were very rare in the TG, whereas some teams in the OG produced results that differed extremely from the original study. Many were over 100% different, and in rare cases, this was more than 500% different (see Project Repository).

### 3.1. Qualitative investigation of error

Grounded in our qualitative investigation of each team’s workflow, we identified six aspects that caused errors in their computational reproduction efforts. These errors were distributed across most teams. As figure 2 shows, roughly 70% of all teams took some action in their workflow that led to results that did not match those of the original study within a 95% confidence interval.

#### 3.1.1. Mistake (29 instances in 24 teams)

These are steps that teams did not consciously intend to take. Nearly all are coding errors, such as copy-pasting the same code snippet over and over and forgetting to alter the variable name, reversing the wave values (1996 instead of 2006), mistakenly recoding all values in a dependent variable to zero, forgetting variables in the analysis or including the wrong countries in the sample. A few were clerical errors where teams reported coefficients instead of odds ratios or pasted the wrong set of results into the result template (see examples in table 1).

**Table 1.** Selected examples of mistakes and their curations.

team	original	curation
5 (Stata)	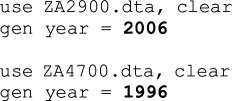	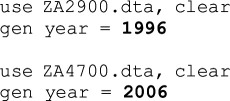
10 (R)	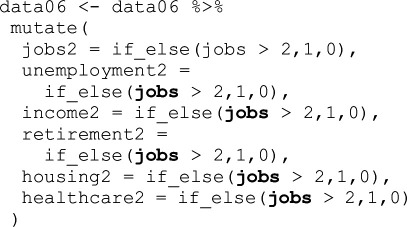	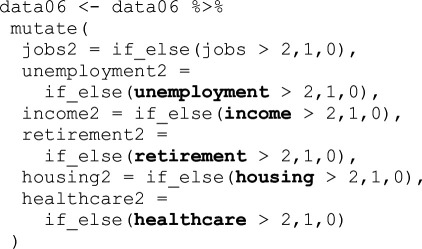
41 (MPlus)	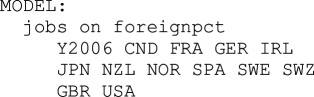	*We did not curate this code because the team noted: ‘they don't mention which country was used as the reference category (we used Australia)’. Therefore, from the team’s perspective this was not a mistake, even though original Stata code was available to demonstrate the US as reference category.*

Note: Grey highlight indicates actual code snippets; **bold** indicates mistakes and curations.

#### 3.1.2. Procedural (62 in 39 teams)

Researchers routinely took slightly different coding steps than the original researchers. We see these steps as ‘procedural’ because we assume this is how the teams always do their research rather than conscious decisions made uniquely in this particular study. For the OG in particular, this type of error reflects the team’s best efforts to reproduce models to which they had no original code and minimal description, presumably left to draw on their own previous procedural experiences. Many procedural errors had to do with socio-economic status variables. For example, some teams coded an employment status of ‘helping a family member’ into ‘not in labor force’ when the original study coded this as ‘part-time work’. Others coded this same variable as ‘unemployed’, and some coded ‘unemployed’ as ‘not in labor force’. Two teams disaggregated this variable into ‘full-’ and ‘part-time’ based on a third variable measuring hours of work per week. In the TG, such departures from the original study in recoding decisions were less common.

The treatment of missing values was also a common source of variation in both groups. Some used listwise deletion on all variables prior to running a regression, some only on all four dependent variables and others removed them uniquely for each model. A peculiar problem arose in some cases where dummy variables were coded with the object of interest as ‘1’ (like ‘in labor force’) and then all others (including true missing values) coded as ‘0’ meaning that values were *added* to the analysis that were dropped in the original study.

Other influential decisions concerned decimal place reporting and software type. After conducting this study, we are acutely aware that *R*’s base programming language uses ‘bankers rounding’ which rounds last decimals of 5 up or down to achieve a mean as close as possible to the original, while *Stata* rounds it up to the next whole decimal. This alone may undermine attempts to find exact replication at two decimal places across software types. Moreover, teams reported varying degrees of precision ranging from 1 to 3 decimal places. Other cases included keeping only the Western German sample and dropping those from the Eastern German sample as representative of Germany. This is a common practice for studies that include data prior to 1990 or analysing data close to 1990; thus, it is arguably procedural. We code a team using SPSS and point-and-click methods for data recoding as procedural; but it also receives a Missing Component categorization if they did not output the code.

#### 3.1.3. Mistake-procedural (22 in 18 teams)

There are cases where we cannot safely conclude that it was a mistake or a product of a research team’s standard approach to working with data as defined above. This category is dominated by recode variations for the socio-economic variables. The TG technically had all the information necessary to recode variables identically to the original study; however, this information was not entirely presented in the manuscript itself and required a careful investigation of the original study’s *Stata* code to fully grasp. Table 2 lists the most common recodes in this and the Procedural category, and table 3 gives some concrete examples. These same differences in recoding socio-economic status are coded ‘Procedural’ for the OG and ‘Mistake-Procedural’ for the TG.

**Table 2.** Common procedural recoding variations in socio-economic status variables.

A	‘helping family member’ coded ‘not in LF’ (was ‘part-time’ in original)
B	‘completed primary’ coded ‘ secondary’ (was ‘primary’ in original)
C	‘incomplete university/tertiary’ coded ‘university’ (was ‘secondary’ in original)
D	‘helping family member’ coded using ‘hours worked per week’ variable to split respondents into either ‘full-time’ or ‘part-time’
E	‘unemployed’ coded as ‘not in LF’
F	‘student’ coded as ‘unemployed’
G	‘housewife/-man, home maker’ coded as ‘unemployed’
H	Recoded ‘none’ or ‘still in school’ as missing on education
I	‘helping family member’ coded as ‘full-time’
J	‘housewife/-man, home maker’ coded as ‘full-time’
K	‘helping family member’ coded as ‘missing’

Note: Non-exhaustive. Letter denotes these coding rules in electronic supplementary material, table S6 in appendix B.

**Table 3.** Examples of procedural categorizations.

source	coding
original ISSP 1996 data codebook (p. 118)	v205<DEGREE > R: EDUCATION II: categories None, still at school Incomplete primary Primary completed Incomplete secondary Secondary completed Incompl + compl.semi-higher + incomplete university University completed
original study (Stata)	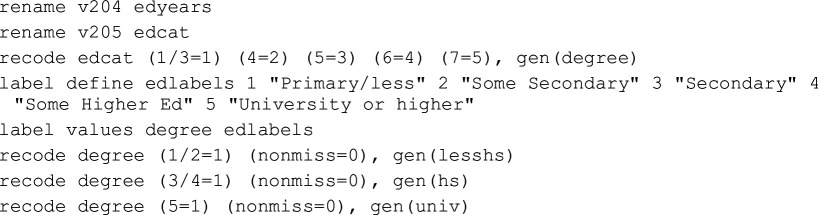
team 39 (Stata)	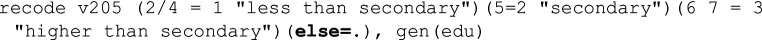
team 83 (R)	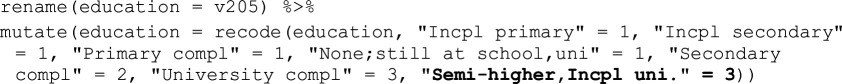

Note: **Bold** are specific sources of error. Top row are the official survey definitions of the variable, second row is the original study’s code and the bottom two rows are examples of errors.

The Mistake-Procedural category also appeared in the OG in cases where more blatant socio-economic status miscoding occurred. One example is Team 84 who recoded ‘less than part time’ as ‘unemployed’ and also ‘other/not in labor force’ as ‘ missing’. Again, this is categorized here because there may be plausible arguments behind these decisions in the minds of the replicators at that time. These and others that were larger deviations than those listed in table 2 were coded as Mistake-Procedural rather than Mistake. In three cases, the teams used some form of robust clustering of standard errors. This was not mentioned in the originally provided Methods section; however, this model feature is a reasonable assumption when asked to design a model with country and wave dummies (i.e. a basic multi-level model).

The top row of table 3 provides the original scoring of the variable v205 from the 1996 wave of ISSP data indicating educational qualification level. The second row is *Stata* code from the original study. Below that are example snippets from two different teams. In the third row, we see that Team 39 did not assign a new value for ‘1’ (‘less-than-high-school’), so it was recoded into ‘.’ (missing in *Stata*). This is not safely categorized as Procedural as the team was in the TG with access to the original code. Then again, only the code, not the Methods section of the article offers this information, so they might have been following the Methods section rather than closely reading the code. Thus, in our categorization scheme, it is Mistake-Procedural. Team 83 recoded those who had incomplete university education as ‘3’ having completed university, instead of ‘2’ indicating secondary education completion (the variable ‘hs’ in the original study). We do not code this as a mistake because the team was in the OG and did not have access to the original code but had to use their best guess, because precise definitions of ‘secondary [education]’ were not provided in the Methods section they were given (see electronic supplementary material, appendix A). By definition, Mistake-Procedural and Procedural are often identical types of error in the code but fall into different categories based on the information available to participants.

#### 3.1.4. Missing component (4 teams)

Four teams had missing parts of their workflow rendering it irreproducible. Some users of *SPSS* did point-and-click recoding and merging of their data, and some teams did data pre-processing that they simply did not save. For these cases, curation is not possible, because asking them to redo their analyses would contradict the ecological goals of the study.

#### 3.1.5. Interpretational (3 teams)

In three cases in the OG, the team interpreted the description of the models in a manner that differed from the original study. Two involved the centring of independent variables. Although the Methods section provided to the OG did not specifically offer instructions for any transformations of continuous variables such as age, it did not mention *not* to take this step either. As it is plausible to interpret a multi-level model as involving centring of some or all continuous variables, this type of error is categorized as interpretational. The third case involved selecting data based on the Methods section statement that ‘all thirteen rich democratic welfare states with data for both waves are included’. The team selected on the provided immigration data, which was available for 17 countries, and then randomly selected 13, whereas our intention was that they select on available individual-level ISSP data for which there are only 13 countries in both waves. As this was not explicit and the team even mentioned this in their workflow notes, we classify this error as interpretational in nature.

#### 3.1.6. Questionable method knowledge (3 teams)

Our call for researchers explicitly asked for basic regression analysis skills, including multi-level modelling. However, three teams demonstrated that they might not possess such skills. In one case, the team admitted to using *Stata* for the first time, which constitutes a lack of software knowledge. In two other cases, the teams analysed the two separate waves of data in separate regressions, rather than a pooled model with dummy variables for country and wave. It is perhaps bad luck, or a result of the less transparent materials, that we have this category, given that both teams who analysed waves separately were in the OG. However, we suspect that it is most likely a lack of experience, because the Method section they were given stated, ‘The ISSP data from 1996 and 2006 are pooled and all thirteen rich democratic welfare states with data for both waves are included […] These models therefore have dummy variables for countries and years’ (see electronic supplementary material, appendix A).

### 3.2. Correlates of errors

Errors were distributed across most teams (figure 2) and about one-third of the variance in whether a model was a successful reproduction or not took place between teams (figure 1). This variance allows us to statistically analyse the sources of uncertainty we found in our three replication outcome measures. This includes attention to the statistical skills and experience of the teams, their perceived difficulty in completing the task and of course the experimental condition itself (transparency of materials). table 4 presents the raw results (electronic supplementary material, tables S8 and S9 in appendix B present curated and trimmed). Pooled results are in the first left column. Although teams using *Stata* had a higher Directional Reproduction rate on average, multivariate analysis adjusting for the potential correlations of other variables suggests very broad confidence intervals (*b* = 0.14, s.e. = 0.08); that is, these data are as likely to be observed if the statistical effect was truly zero. Statistics skills and studying or having acquired a sociology degree appear to have no association, neither a sizeable coefficient nor a rejected null hypothesis. Teams reporting that the task was more difficult were less likely to succeed (failed NHST; *b* = −0 .14, s.e. = 0.05, *x*-standardized). Finally, teams were roughly 25% more likely to succeed if they were in the TG (*b* = 0.25, s.e. = 0.06 , *x* dichotomous) all else equal. We place no interpretational weight on coefficients that are both small and lack a *p*-value below 0.05. The adjusted *R*-square suggests that we can explain about 25% of the team-level variation in results. Group-specific results suggest that *Stata* users were far more likely to directionally reproduce than non-*Stata* users in the TG, but this effect was absent in the OG. Higher perceived difficulty is also associated with lower reproducibility in both groups, and given its correlation with statistics skills, we assume that it absorbs the effects. The experimental condition, perceived difficulty and statistics skills are all endogenous. These results are similar for Exact Replication, although far less variance is explained and effect sizes are also smaller. It is important to note that a low-N weakens our capacity for statistical inference in group-level analyses. Our pre-registered power analysis was only designed to detect an experimental group difference, not to detect multivariate effects, so this analysis should be considered exploratory.

**Table 4.** Multivariate analysis of computational reproducibility in 85 teams, raw results.

	directional reproduction			exact replication			replication error		
variable	pooled	TG	OG	pooled	TG	OG	pooled	TG	OG
(intercept)	0.45^***^ (0.06)	0.60^***^ (0.08)	0.51^***^ (0.07)	0.89^***^ (0.03)	0.91^***^ (0.03)	0.91^***^ (0.04)	0.44 (0.23)	0.03^***^ (0.01)	0.56 (0.36)
Stata	0.14 (0.08)	0.24^*^ (0.10)	0.01 (0.09)	0.04 (0.04)	0.07^*^ (0.03)	−0 .01 (0.06)	−0 .19 (0.27)	−0 .02^*^ (0.01)	−0 .33 (0.47)
stat-skill	−0 .03 (0.02)	−0 .06 (0.03)	−0 .00 (0.03)	−0 .01 (0.01)	−0 .02 (0.01)	−0 .01 (0.02)	−0 .07 (0.07)	0.00 (0.00)	−0 .14 (0.14)
difficult	−0 .14^**^ (0.05)	−0 .17^*^ (0.08)	−0 .13^*^ (0.05)	−0 .06^*^ (0.02)	−0 .04 (0.03)	−0 .08^*^ (0.03)	−0 .01 (0.02)	0.01 (0.01)	−0 .02 (0.04)
sociology degree	−0 .03 (0.07)			−0 .02 (0.03)			0.01 (0.04)		
TG	0.25^***^ (0.06)			0.05 (0.03)			−0 .06 (0.03)		
observations	85	39	46	85	39	46	85	39	46
*R* ^2^	0.297	0.265	0.164	0.163	0.214	0.138	0.050	0.267	0.049
*R*^2^ adjusted	0.252	0.202	0.104	0.110	0.147	0.077	0.000	0.205	0.000

Note: Unstandardized OLS regression coefficients predicting outcomes aggregated to their mean by team; standard errors in parentheses. Degree omitted from group-specific regressions due to low predictive power and smaller sample sizes. TG = transparent group with access to all materials and OG = opaque group with no code and less methodological information.

*p *<* 0.05, **p *<* 0.01, ***p *<* 0.001

What we mostly cannot explain is the degree of error present on average per team. Regressions on Replication Error yield adjusted *R*-squared of zero for the pooled sample and the OG. The TG regression produces results with an adjusted *R*-squared of 0.21 (roughly 21% variance explained), and this seems entirely attributable to the tiny effect of using Stata*,* which on average is associated with a 2% lower error margin; this might be, for example, the difference between an exact replication (error = 0) and an odds ratio that is 2% larger or smaller than the original.

It seems clear that transparent materials are a cause of replication success likelihood. However, there is a significant negative Pearson correlation of Stata with Difficult in both groups (*r* = −0.17 TG, *r* = −0.39 OG; see electronic supplementary material, table S11, appendix B). With such low case numbers, we are unlikely to be able to adjudicate clearly between these two variables. We note that the signs for both mostly pattern as expected despite wider confidence intervals.

Turning to the trimmed and curated results, some statistical associations remain similar. However, curation rendered the TG to have very little explained variance and no significant coefficients for all three replication outcome measures. This may relate to the fact that after curation, 98.2% of the variable Directional Reproduction are zeros, leaving little variance to explain. This is not the case for the trimmed data where we see a high *R*-squared. More striking in the curation is a much higher degree of explained variance in the OG. We attribute this to the curation of *major* mistakes, which we assume are somewhat random, and once we remove them, we are left with clearer associations between perceived difficulty of the task and the accuracy of the outcome. If the curated results are more ecologically valid than the raw results, we would conclude that error is a product of the researcher’s abilities and challenges encountered in their research, whereas the raw results suggest that error is mostly random if they lack access to the original code.

Finally, we investigate our qualitative categories Mistake and Procedural using regression analyses. For the Mistake outcome, we include only those teams that had at least one instance of the category Mistake or Mistake-Procedural (= 1) and compare them to all other teams (= 0), with those having only Procedural being dropped. For the Procedural outcome, we reverse this and drop teams with any Mistake. Respectively, we drop teams with any Procedural or Mistake errors from the analyses because we want to isolate the likelihood of committing Mistake or Procedural errors relative to not otherwise making errors.

Table 5 shows that the perceived difficulty of the replication, and being in the OG led to a much higher likelihood of Mistake and Procedural errors alike. Keeping in mind the high correlation of *Stata* and *Difficult*, it is unsurprising that the *p*-values are above our cutoffs when we run group-specific regressions. We provide *p*-values owing to convention, but again do not place a strong stake in them given sample sizes and a lack of pre-registration. The explained variance in the TG is high and driven mostly by whether the team used *Stata* or not. The effect of Difficult is the only variable that mathematically explains variance in the OG, but confidence intervals still overlap zero even if we drop to 90% confidence, and overall, the regression explains very little variance. We conclude that both Mistakes and Procedural errors are more likely if researchers face greater subjective difficulty in completing their replication tasks, regardless of the transparency of materials. As we doubt that *Stata* users are more or less skilled than *R* users, we speculate that the correlation of *Stata* and Difficult might result from *Stata* users having access to the code; however, when they do not, they might be either better trained or have more experience at this stage in history, because *R* is much newer in social science.

**Table 5.** Multivariate analysis predicting qualitatively categorized sources of error.

	mistake			procedural		
	pooled	TG	OG	pooled	TG	OG
(intercept)	0.67^***^ (0.10)	0.51^***^ (0.11)	0.58^***^ (0.14)	0.63^***^ (0.10)	0.42^***^ (0.09)	0.51^**^ (0.15^)^
Stata	−0 .14 (0.11)	−0 .31^*^ (0.13)	0.01 (0.18)	−0 .07 (0.11)	−0 .29^*^ (0.10)	0.13 (0.20)
stat skill	0.05 (0.03)	0.10^**^ (0.04)	−0 .02 (0.05)	0.05 (0.03)	0.10^**^ (0.03)	−0 .01 (0.06)
difficult	0.20^**^ (0.07)	0.28^**^ (0.10)	0.16 (0.10)	0.22^**^ (0.08)	0.35^***^ (0.08)	0.21 (0.12)
exp	−0 .29^**^ (0.10)			−0 .38^**^ (0.10)		
observations	77	38	39	72	34	38
*R* ^2^	0.240	0.366	0.102	0.291	0.543	0.086
*R*^2^ adjusted	0.198	0.310	0.026	0.249	0.497	0.006

Note: Linear probability models. Unstandardized OLS regression coefficients predicting outcomes aggregated to their mean by team; standard errors in parentheses. TG = transparent group with access to all materials and OG = opaque group with no code and less methodological information.

*p < 0.05, **p < 0.01, ***p < 0.001

## 4. Discussion

When attempting to reproduce the numerical results of a published study, different replicators obtained different results with varying sources of error. The error rate was exacerbated when there was less transparency. In other words, less information about data preparation and analytical choices is available to them. We see no reason to assume our sample is any more or less technically capable of replication than the population of all social and behavioural scientists; however, it is admittedly plausible. If we nonetheless hold to our assumption that they are representative of social science researchers using computationally intensive hierarchical secondary data analysis, then we face a reality where it might take more than one independent attempt to produce reliable reproduction results. Averting this reality requires much higher standards of transparency and possibly lower requirements of precision in numerical computational replication.

**Figure 3.**
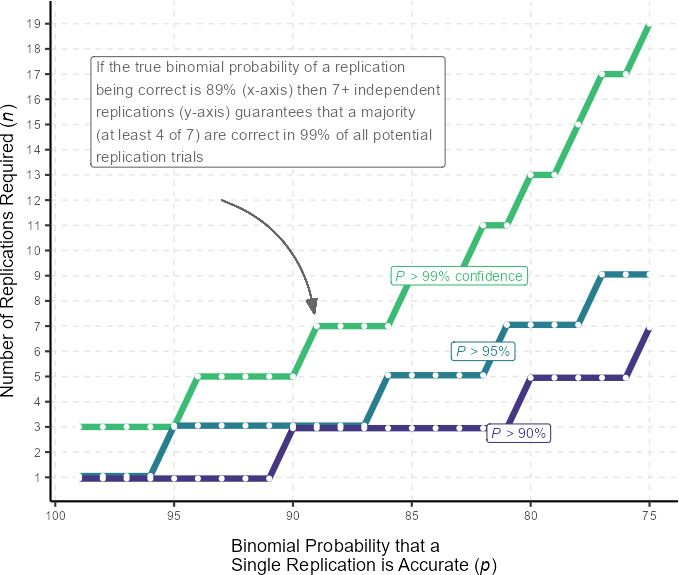
Simulation of the number of independent replications required to achieve a majority correct at different *P* cutoffs

We would hope that replicators communicate with the original authors when they do not arrive at the same results [26]. But sometimes such communication is not possible. As a thought exercise, let us assume that what we mean by reliability is 95% confidence that the conclusion indicated by a majority of the observed number of replications matchs the ‘correct’ conclusion. This would mean that for a single replication to suffice, it would have to be correct at least 95% of the time. If there is a lower success rate, then more than one replication is necessary to reach the 95% threshold. We simulate this problem by plotting binomial probabilities of researchers coming to a correct computational replication by the number of replications needed to achieve a certain critical probability (figure 3).

In real-world research, we do not know in advance what is ‘correct’ or ‘ true’, as this would undermine the need to conduct research in the first place. This is why computational reproduction is an important test case, as we *can* know the correct result in advance, at least within a degree of rounding error. We assign 51% of *n* replications arriving at *x*, the correct answer, as our minimum definition of majority. Thus, in one replication, x must be equal to 1; in two replications, x = 2; in three replications, x ≥ 2; in four replications, x ≥ 3 and so forth. We can then calculate the probability *P* of arriving at *x* successful outcomes among *n* replications when the true likelihood of a successful replication as *p* using the Bernoulli trials formula $P\left(X\geq x\right)=f\left(x\right)={(}_{x}^{n}{)p}^{x}{\left(1-p\right)}^{n-x}$. Then we can calculate the minimum value of *n* for our critical threshold of *p* = 0.95 (i.e. 95% confidence), and this tells us how many independent replications it takes to achieve reliability at different theoretical values of *p* replicability. Figure 3 presents different values of *n* at theoretical values of *p* at different *P* thresholds.

Figure 3 is a simulation, and *p* is a mostly unknown property of replications, but if we use the pooled directional reproduction results of this study (92.5%) as a potential value for *p*, then it would take at least three independent replicators to achieve reliability, when this is defined as a majority of replications verifying a correct result 95% of the time. We encourage use of the pooled rate as our current best approximation because it lies between two extremes of transparency, possibly something closer to reality. If we demand 95% of within-team effects be directional reproductions, then it would take more than 10 replications per study because less than 70% of teams could achieve such three-way reliability—a binomial probability of a majority of replications, 95% of the time, and 95% of within-team effects verified. This may sound extreme, but looking back at our literature review, using a 92.5% value for *p* is generous when compared to other studies which tended to have it between 50 and 75% [17–19].

## 5. Conclusion

How reliable are computational reproductions? Our study makes clear that the answer to this question depends on several factors. Overall, the rate of directional reproduction of the original results appears reliable at 92.4%, but does not necessarily meet a common standard alpha of 95%. Only when curating mistakes made by the replication teams is the 95% threshold crossed. We uncovered much about the reasons for uncertainty in computational replication. The most important is transparency. A successful single computational reproduction of a given model is far more likely if the complete, well-annotated original code is available. The rate of directional reproduction that the results are the same direction and sign in our experiment was 95.7% for the TG, noticeably higher than the OG at 89.2%, which received less information about the original study’s methods (see figure 1 ). The precision of the replication demanded also matters. When demanding exact numerical replication within 1% of the original odds ratio, the TG success rate was nearly 30 percentage points higher (77.4 versus 50.1%). The skills of the researcher and the subjective difficulty of the replication are also important. In our sample of researchers, we could not reliably disentangle these two factors given their correlation (at around −0 .3) and that the experimental condition gave a seemingly more difficult task to one group (the OG); however, a one s.d. more difficult replication experience reduced the likelihood of replication by anywhere from 5 to 15% depending on the demanded precision and handling of outliers. Finally, if the replicator is ‘ fluent’ in the same software as the original study, this increases the likelihood of a successful replication by 7 to 24%, towards the higher end of the distribution with more transparent materials (see table 4 and electronic supplementary material, tables S8 and S9). Crucially, the treatment effect of our experiment is shown to hold strong after adjusting for other aspects of the replication teams.

In our example, two of the PIs independently replicated the original study’s effects within two decimal places in both *Stata* and *R*. Their goal was to come to this result, and the one working in *R* spent several hours getting to this point. This meant that there was a very high degree of motivation to arrive at these results that may not have been present in the participants of this study, given that they were promised publication as long as they completed all tasks assigned to them, regardless of their outcomes. Although they were asked to approach this study as they would their usual work, our study might not exhibit ideal ecological validity. We tried to control for less motivated or less skilled outliers by offering curated and trimmed sets of results. These steps increased the pooled directional reproduction averages to 95.2 and 94.1% respectively, while the exact replication rate averages came up to 71.7 and 66.4%. The highest group-specific exact replication rate was only 83.9% in the TG in the curated set of results. This is a level that is much lower than the 90 or 95% cut-offoften often used as a standard for a result to be considered reliable.

The studies we reviewed in §1 suggest computational reproducibility might lie between 50 and 75% of results within and between studies. Thus, our finding looks promising in this light. However, we need to be cautious about how we define reproduction. If we defined it at the team-level rather than model-by-model, demanding that at least 95% of all results within a given team are without error is a necessary condition for a ‘successful’ replication, this yields only a 64.9% success rate (see figure 1 ). This means that most teams had at least a few models that failed to replicate. Most teams had errors. Only 14 teams had 100% exact numerical replication, all from the group with transparent access to the original code. This may owe to another factor that we uncovered in our qualitative coding of the teams’ workflows. We discovered that there was *information in the code of the original study that is necessary to achieve an exact replication that otherwise cannot be found in the paper or supplementary materials*. This was most evident in minute details relating to the recoding of socio-economic status variables. In this study, like many in the social sciences, socio-economic status was a key adjustment variable, and its construction by the original authors required combining responses from various questions from the original survey data (see table 2). If potential replicators do not have access to or use the same coding language as the original, it is difficult for them to understand all steps in the code. This means that *Stata* users in the TG had the easiest access to additional, but crucial, methodological information about the study. An argument for more transparency, if not clearer presentation of methods, in future studies.

Without reference to any numeric reproducibility rates, our qualitative investigation shows there is analytic flexibility, or what Gelman & Loken [27] refer to as ‘researcher degrees of freedom’ leading them through a garden of forking paths, *even in research so narrow as a computational reproduction*. This surely is the case in the TG who were provided with the original analytical code. Gary King [28] refers to provision of original code as the most elegant way to engage in reproducible research. Our results demonstrate that truly elegant code should be well-structured, well-documented and comprehensible for researchers who do not use the software, but even so, might still leave replicators subject to uncertainty. Our results suggest that this uncertainty may be procedural, as in, idiosyncratic to the researchers or their research processes. We observed uncertainty in results that were not based on conscious analytical decisions or mistakes but occurred as the teams engaged in their standard idiosyncratic research practices and previously learned software routines.

There are three clear implications of our findings. The first is that transparency is critical to increasing the reliability of science. We understand science to mean repeated testing of scientific claims to form a set of results that can be trusted as communicating accurate information about the world [29]. Like many social and behavioural science journals, we did not control the research process of researchers. We asked them to do work as they normally do and then submit their findings. This suggests that institutions, journals and teachers should place much higher quality demands on scientists, because their transparency behaviours are far from ideal [10,30,31]. Our findings underscore that we would immediately make social and behavioural science far more reliable: if all journals not only required but checked code. Today some journals hire third parties to provide code checking, but not all journals are in a financial position to do this. Therefore, we suggest that one reviewer, or elected person per paper, provides a computational reproducibility check, similar to what the AJPS started in 2015 and what *Psychological Science* recently adopted [32].

Second, we conclude that transparency is not a cure-all. If the quality of transparent materials is lower, researchers might be forced to make assumptions that change the results. This was clear from our qualitative investigation where teams lacking specific step-by-step instructions about how to recode variables made different ‘guesses’ along the way. Moreover, there is an assumption made by many researchers that the gold standard for transparency should be a ‘push-button’ replication. Our own research and knowledge gained from this study suggest that this is an oversimplification. If a researcher does not know how to use the software needed for a ‘push-button’ replication, they cannot run it. For example, a truly push-button replication might require installation of *Python*, *R*, *Stata* and/or *Jupyter Notebooks*, plus the skills to get them working properly with one another on any given operating system and version; a good example of push-button replication that requires such skills and installations is the *Jupyter Notebook* of a study by Connelly & Gayle, which is perfectly reproducible but only via multiple software installations and the knowledge required to use them [33]. Also, differences between operating systems, packages and processors can produce different results [34] like with the different *Stata* versus *R* rounding defaults. In this study, replication teams submitted their results with different numbers of decimals, and this may have been a product of software or package defaults. Default settings often change across versions or software packages. Thus, the ‘tacit knowledge’ that researchers require to execute their studies ‘cannot be fully explicated or absolutely established’ in practice [35].

Related to this point, if the data cannot be shared and must be sought out by the replicator, this introduces potential error in a supposed ‘push-button’ replication. Data in repositories often change over time, and occasionally archivists make these changes without version control [36]. Remedies for this point are at least twofold. Improved teaching of the many potential pitfalls in scientific reproducibility and replication as a methodology, even as early as during undergraduate studies, would reduce errors and the perceived difficulty of reproduction tasks [37]. It would both increase attention to detail and awareness of key aspects to producing transparent and reliable work [1,38]. Expecting knowledge of virtual computing environments might be overly optimistic, but at least sharing of environment and dependencies within the software (sometimes known as a ‘colophon’ or ‘session info’) theoretically gives all necessary information to remove intra-software and package variation in results. This could be part of teaching programs for cutting-edge analytical pipelines and replicable research [26,39]. In fairness, many journals are grappling with this exact issue and can be commended for pushing to adopt code-sharing and ideally push-button replications [40,41]. Not only are transparency and replication policies difficult to develop given the challenges in creating something like a push button replication, but clarifying and enforcing them adds an additional layer to the problem [42]. We certainly support efforts to produce push-button replications as they can be checked by at least those with requisite knowledge, for example, how to implement older versions of software and packages. An alternative solution is that scholars themselves can produce reproducible research via a third-party platform such as Colab or Code Ocean, where users can push-button run the code in the virtual computing environment. These also can have a DOI to make for easy linking. There are no quality controls or guarantees that these are long-term viable, but they are an excellent option when there are no other alternatives.

Finally, our study implies that we should be humbler and more cautious in our communication of science than currently practised. Social and behavioural science, like all science, may not be as reliable as previously thought. We have shown here that this may be true even in a task as supposedly decision-free as computational reproduction. Looking back at our simulation in figure 3, we will likely often need multiple replications to achieve reliable knowledge production, which in many contexts will not be a viable option. This need not contribute to a ‘crisis’ narrative but rather a reminder to continuously improve our work and the institutions supporting it, in particular the journals, their policies and the level of enforcement. It is, after all, our job as scientists to measure and communicate uncertainty. Therefore and nonetheless, reliance on a single model, or reporting results as definitive or absolutely truthful based on a single reproduction is irresponsible, misleading and contrary to the scientific method.

Here our study links to analogous evidence of inter-researcher variability when researchers conduct similar original research tasks as seen in ‘many analysts’ and multi-analyst studies [14,43–46]. It is difficult to test how well these findings generalize beyond simple computational reproduction, given the challenge of obtaining a reliable prior probability of coming to a correct result in any given study. We do not know if an original study is ‘correct’, mistake-free or using the most theoretically plausible data-generating models [47]. Prediction markets or *z*-curves are suggested options to estimate plausible expected replicability rates [48,49], but any attempt to identify a ‘true’ replicability rate can quickly digress into a philosophical debate regarding the nature of truth.

Our study is not without limitations. It is possible that the peculiarities of a task involving ISSP data with a 10-category employment variable and a 7-category education variable (at least in the 1996 wave) made researchers especially prone to procedural variability. However, we note that macro-comparative social scientists do a great deal of survey-based research, and most surveys generate data on ISCO codes, education categories (that often vary by country) and several labour market statuses that are not always consistent (like respondents reporting being ‘unemployed’ in one question and working ‘part-time’ in another). Moreover, a study with odds ratios as an estimand may have peculiarities. Logistic regression involves transformation from linear to logit, and then again the coefficients are transformed into odds ratios. This leaves more steps for error. Moreover, logistic regression is iterative rather than definitive, and there are various ways that a researcher can engage in iteratively arriving at the best underlying linear combination, unlike ordinary least squares.

We are also limited in our ability to draw a population inference from our sample. There is no straightforward way to define the global population of potential replicators. Our sample-N at the team level split into two groups does not give substantial statistical power, only at the effect level as pre-registered; therefore, we must be cautious about inference at this level. We invited anyone who has working knowledge of multi-level modelling and experience with survey and country-indicator data to join, yet not all had experience in the topical area and some may not have accurately read or met the qualifications. Follow-up studies should consider limiting participation to experts on the topic, a particular discipline or other criteria designed to eliminate additional noise likely generated in our effort to measure inter-researcher reliability.

In our study, the opaque group attempted to replicate under exceptionally intransparent conditions, without code and without even numerical results. It thus does not come as a surprise that this group was far less likely to reproduce the original study. This is an extreme case, and we do not expect studies to be published without their numerical results. Yet it is fair in the sense that many studies offer footnoted ‘additional analyses’ which often support the findings of the main analyses without numerical evidence. Moreover, we know that researchers might report false numerical results [50,51], which is a case where any replication attempt without also having the code is essentially a new study. Our study makes loud and clear the fact that when original code is not available, replication error rates increase markedly. Transparency is a low-cost alternative to larger-scale methods of ‘stabilizing’ estimate uncertainty like the crowdsourced replication we conducted or even larger *Metaketa* efforts [52]. If their goal is the efficient and reliable production of collective knowledge, a social scientist should not need ethical rules or enforcement mechanisms to want to generate and share high quality code. Their motivation should only increase when informed about the costs of errors and potentially false claims against their work, not to mention reliability. This same logic applies to social science journals when writing up and enforcing transparency policies. It is true for all of science.

## Data Availability

We provide all data and workflow on GitHub. For our last submission, the authors had problems accessing Zenodo; therefore, to ensure scientific reliability, we can no longer trust Zenodo in the peer review process. Here is our GitHub link for our entire reproducible repository [53]. Supplementary material is available online [54].

## Footnotes

^1^
Calculated from their own figure 1.

^2^
GitHub Project Repository: https://github.com/nbreznau/how_many_replicators.

^3^
Although the OG condition may seem extreme, we are aware of many published studies in which authors claim they conducted additional analyses with similar findings without offering any numerical results or code for these.

^4^
This served the following two purposes: (i) it gave the authors some opportunity to make ecologically valid mistakes, and (ii) the original code contained far more variable constructions and models than were of interest to this study; thus a fresh write-up served to reduce confusion in the code submitted by the replication teams.

^5^
This refers to their main results in their original tables 4 and 5, which readers can view in our workflow file 01_Data_Prep.html.

^6^
In two cases, further exchanges with the teams were necessary to get their code running because it contained procedural elements the PIs were not familiar with— once in an R project and another running MLwiN in a Stata shell.

^7^
For example, 0.5 and 2 represent identical changes in odds (1/2 and 2/1, respectively) but are different numerical distances from the null odds of one.

^8^
Based on teaching, publications and methods usage; see confirmatory factor analysis in Project Repository.

^9^
The study had a second phase asking the participants to design their own ideal models for testing the same hypothesis as Brady and Finnigan.

^10^
Also available on Github as appendix C.
